# Patient‐Derived Organoids in Functional Precision Oncology: Tumor Modeling, Drug‐Response Prediction, and Clinical Translation

**DOI:** 10.1002/cbf.70321

**Published:** 2026-10-19

**Authors:** Abdullah Jabri, Mohamed Alsharif, Abdulaziz Mhannayeh, Bader Taftafa, Dania Sibai, Abdulrahman Elsalti, Raghad Alsharif, Tanveer Ahmad Mir, Mohammed Imran Khan, Jahan Salma, Ahmed Yaqinuddin

**Affiliations:** ^1^ College of Medicine, Alfaisal University Riyadh Saudi Arabia; ^2^ Department of Internal Medicine Acıbadem MAA University Istanbul Turkey; ^3^ College of Medicine, AlMaarefa University Riyadh Saudi Arabia; ^4^ King Faisal Specialist Hospital and Research Center Riyadh Saudi Arabia; ^5^ King Faisal Specialist Hospital and Research Center Jeddah Saudi Arabia

**Keywords:** cancer organoids, clinical translation, CRISPR functional genomics, drug response prediction, functional precision oncology, organ‐on‐chip, patient‐derived organoids, three‐dimensional cell culture, tumor heterogeneity, tumor microenvironment

## Abstract

Patient‐derived organoids (PDOs) have emerged as an effective experimental platform for bridging the long‐standing gap between traditional in vitro cancer models and the biological complexity of actual tumors. These three‐dimensional cultures (3D), derived directly from patient tissues, preserve essential architectural, genomic, and phenotypic characteristics of their tumors of origin, enabling more accurate modeling of tumor heterogeneity and disease evolution. Over the last decade, rapid methodological advances in organoid culture systems, extracellular matrices (ECMs), and growth factor optimization have facilitated the establishment of organoid models for a wide range of malignancies and the development of large living biobanks representing diverse patient populations. These models are increasingly being utilized to study cancer biology, elucidate mechanisms of treatment resistance, and evaluate patient‐specific therapeutic responses, highlighting their potential as tools for precision oncology. However, important challenges remain, including incomplete representation of the tumor microenvironment (TME), variability in culture conditions, and limited scalability, and the need for greater standardization and validation. Consequently, although PDOs show considerable promise for translational research and personalized medicine, their routine clinical implementation remains largely investigational, and their clinical utility has not yet been fully established. This review examines the technological foundations of PDO generation, assesses their role as experimental models of tumor biology, and discusses their expanding applications in translational cancer research and precision oncology while highlighting key challenges that must be addressed before their widespread clinical adoption.

## Significance Statement

Patient‐derived organoids are increasingly used to study cancer outside the patient, but their potential is often discussed more broadly than the clinical evidence currently allows. This review brings together the methodological, biological, and translational evidence to distinguish what PDOs can already accomplish from what remains experimental. In particular, we highlight an important transition from retrospective demonstrations that organoid drug responses resemble patient responses toward the more demanding question of whether treatment‐naïve PDOs can prospectively guide therapy before treatment is given. We also identify practical barriers that are often underestimated, including limited biopsy availability, variable establishment success, lengthy culture timelines, extracellular‐matrix variability, incomplete immune and stromal representation, and the need for validated clinical response thresholds. By integrating these considerations with emerging approaches such as immune co‐culture, CRISPR functional genomics, engineered biomaterials, and organ‐on‐chip systems, this review provides a framework for assessing when PDO results are sufficiently reliable to support translational studies and when they should remain investigational. The central implication is that the future clinical value of PDOs will depend less on demonstrating that they can reproduce tumor biology and more on proving, prospectively and reproducibly, that their results improve therapeutic decision‐making and patient outcomes.

## 1 ⁠Introduction

Modeling human diseases in experimental systems that accurately recapitulate the complexity of native tissues remains a major challenge in biomedical research. Traditional two‐dimensional (2D) cell cultures, although widely used, often fail to reproduce the three‐dimensional (3D) architecture, cellular interactions, and biological characteristics of human tissues, thereby limiting their ability to predict disease behavior and treatment responses in vivo. In contrast, 3D culture systems have emerged as more physiologically relevant models that better recapitulate tissue structure and function [1]. Among the advancements, organoid technology has become a powerful platform for studying human development and disease. Organoids are 3D, self‐organizing cellular structures derived from stem cells or primary tissues that can replicate vital structural and functional characteristics of their tissue of origin. Because these models preserve important aspects of tissue architecture and cellular composition, they have become valuable experimental platforms for investigating disease mechanisms and evaluating therapeutic responses across several biological domains, including immunology, regenerative medicine, and oncology [2]. Building on these advances, patient‐derived organoids (PDOs) have emerged as particularly promising models for reproducing patient‐specific tumor biology directly from clinical specimens. PDOs can retain important molecular, genetic, and phenotypic properties of the original tumors while allowing for long‐term expansion and experimental manipulation in vitro. Furthermore, PDO models have been found to mimic patient‐specific responses to anticancer treatments, supporting their potential as platforms for drug screening and functional precision oncology [3]. Despite rapid progress in organoid technology and the increasing number of studies utilizing PDOs, several important obstacles remain in terms of standardization, scalability, and integration into translational and clinical research. In addition, the expanding applications of PDOs, from disease modeling and functional genomics to drug development and customized therapy, has necessitated a comprehensive synthesis of current advancements and remaining limits. This review provides an updated overview of PDO technologies, with an emphasis on their methodological foundations, applications in cancer biology and therapeutic response modeling, and increasing significance in precision medicine. We also critically examine the biological, technical, and translational challenges that continue to limit broader clinical implementation. Finally, we discuss future directions that may facilitate the integration of PDOs into biomedical research and personalized cancer care.

## 2 Methods

This review was conducted as a narrative review. A comprehensive literature search was performed using PubMed/MEDLINE, Scopus, and Google Scholar to identify relevant studies published from 2009 to March 2026. Search terms included combinations of “patient‐derived organoids,” “cancer organoids,” “precision oncology,” “drug screening,” “functional precision oncology,” “tumor microenvironment,” “organoid biobank,” “personalized medicine,” “patient‐derived xenograft‐derived organoids,” and “organoids‐on‐a‐chip.” Additional articles were identified through manual screening of the reference lists of relevant publications. Original research articles, clinical studies, methodological papers, and high‐quality review articles addressing the development, biological applications, translational potential, and clinical implementation of patient‐derived organoids across multiple cancer types were considered for inclusion. Priority was given to landmark studies, recent advances, and publications with significant methodological or clinical relevance. Conference abstracts, non‐English publications, and studies lacking sufficient methodological detail or direct relevance to the scope of this review were excluded. As this is a narrative review, no formal systematic review protocol or quantitative evidence synthesis was performed.

## 3 Historical and Technical Foundations of PDO Technology

The roots of modern PDO technology can be traced to the landmark study by Sato et al. [4] that showed single Lgr5+ intestinal stem cells could self‐organize into crypt‐villus structures when cultured within an extracellular matrix (ECM) with appropriate growth factors. This discovery laid the foundation for the widespread adoption of organoid technology across a range of cancer types, essentially offering a novel way to model human cancers in vitro [5]. In contrast to the conventional 2D cell cultures, which poorly mimic the characteristics of cancer patients, these organoids offer a more realistic 3D model that not only preserves tissue architecture but also captures intratumoral cellular heterogeneity [6, 7, 8].

Several key advances have enabled the development of these systems. 3D culture platforms allow cells to develop the proper polarity, cell to cell junctions and tissue‐like structure that are not present in flat monolayer cultures [5, 9]. More complex organoid systems often require specialized culture platforms, such as spinning bioreactors, which offer the required mechanical support and nutrient perfusion [10]. ECM scaffolds such as Matrigel provide structural support for organoid growth by supplying laminin, collagen IV, and additional matrix proteins and growth factors that facilitate epithelial organization and proliferation [11]. However, because Matrigel is derived from murine sarcoma, significant batch‐to‐batch variability can affect experimental reproducibility, and the optimal ECM composition remains incompletely defined for many tumor types [7, 9]. Growth factor supplementation is equally important for maintaining organoid viability and stem cell function. Typical culture media contain activators of Wnt signaling, epidermal growth factor (EGF), and inhibitors of transforming growth factor‐β (TGF‐β) and bone morphogenetic protein (BMP) signaling pathways [11]. For intestinal organoids, EGF, Noggin, and R‐spondin‐1 constitute core components of standard culture media. Selective withdrawal of EGF has also been used experimentally to enrich RAS‐mutant tumor populations, demonstrating how culture conditions may impose selective pressures that resemble aspects of tumor evolution in vivo [7].

PDOs can be established from several tissue sources, each offering distinct advantages and limitations. Endoscopic or needle biopsy specimens are clinically accessible and therefore particularly relevant for precision oncology; however, limited tissue volume and low tumor cellularity may reduce organoid establishment rates [12]. Success rates vary considerably according to tumor type, with rectal cancer biopsies generally demonstrating higher establishment rates than head and neck cancers [12, 13]. Another limitation of biopsy‐derived cultures is the preferential expansion of normal epithelial cells, which may outcompete malignant cells during organoid establishment, as has been observed in airway epithelial cultures [14]. Surgically resected specimens generally provide larger quantities of high‐quality tissue, facilitating organoid establishment and the development of living biobanks across multiple tumor types [10, 13, 15, 16]. For example, colorectal liver metastasis‐derived organoids have demonstrated high establishment success while preserving the histopathological and molecular characteristics of their tumors of origin [15]. However, reliance on surgical specimens inherently limits representation to patients who are eligible for surgical resection, introducing potential selection bias [10]. Malignant effusions represent another promising source of tumor cells. Cells isolated from pleural or peritoneal effusions can be obtained using minimally invasive procedures, frequently demonstrate high organoid establishment rates, and enable investigation of metastatic heterogeneity across multiple tumor sites [14].

Once established, PDOs require serial expansion through mechanical or enzymatic passaging. Depending on tumor type and culture conditions, organoid generation and expansion typically require several weeks to approximately 3 months, although optimized protocols have reported successful drug testing within 4 weeks in selected settings [11, 12].

## 4 PDOs as a Model of Cancer Biology

### 4.1 Recapitulation of Tumor Architecture, Heterogeneity, and Tumor Microenvironment (TME)

The way we model human cancer in vitro has changed considerably over the past decade and PDOs are not an exception. Instead of compelling tumor cells to flatten and adapt to monolayer growth, organoid systems allow them to expand within a 3D ECM that more closely resembles the structural context of the tissue they were derived from. In epithelial malignancies, in particular, this often results in preservation of glandular organization, apical and basal polarity, and differentiation states that mirror those observed in the primary tumor [17, 18, 19, 20]. This architectural preservation is not merely descriptive. The physical arrangement of cells influences signaling gradients, nutrient diffusion, oxygen availability, and intercellular communication. When tumor cells are removed from this spatial context, many of these interactions are disrupted. Organoid culture restores part of that microanatomical framework, which helps maintain cellular behaviors that are frequently altered in conventional 2D systems [18, 21].

Tumor heterogeneity is another defining feature of cancer biology, and it remains one of the strongest arguments for organoid use. Tumors evolve as ecosystems composed of genetically distinct subclones, each with variable growth properties and therapeutic sensitivities across different cancer types studied using patient‐derived organoid models (Table 1a). Studies across colorectal, breast, ovarian, liver, and pancreatic cancers consistently demonstrate that organoids preserve interpatient diversity and, to a meaningful extent, intratumoral heterogeneity [3, 17, 19, 22, 23, 24]. In colorectal cancer (CRC), large prospective organoid biobanks retain chromosomal instability patterns and mutational landscapes that reflect those of the parental tumors [17]. Single‐cell analyses further suggest that subclonal populations present in primary tissue can persist in culture, allowing investigators to explore clonal dynamics under controlled experimental conditions [24]. It is important, however, not to overstate this preservation. Any in vitro system imposes selective pressures. Some subclones may expand preferentially over time, particularly with extended passaging. For this reason, early passage validation and careful experimental timing remain essential when interpreting clonal behavior in organoids.

**Table 1a:** Methodological framework for PDO‐based drug testing across cancer types.

Cancer type	Sample source	Establishment rate	Time to successful culture	Assay platform	Drug‐exposure duration	Viability/readout method	Response metric	Threshold definition	Clinical comparator	Validation status
Colorectal cancer (mCRC) [25, 26]	18‐gauge biopsy of a metastatic lesion (some surgical/FNB specimens in the earlier optimization cohort)	52% overall (107/205); increased from 22% to 75% between 2018 and 2023	Median 61 days (range, 22–166) to a biobank‐ready culture, plus approximately 2 additional weeks for screening; 7–25 passages (median, 15)	Seven‐drug panel (5‐FU, oxaliplatin, SN‐38, 5‐FU+oxaliplatin, 5‐FU+SN‐38, trifluridine/tipiracil, panitumumab); CyQUANT, 384‐well format	5 days	CyQUANT (nuclear fluorescence/DNA content)	GR_a_ᴜᴄ (area under the growth‐rate‐inhibition curve)	Normalized GR_a_ᴜᴄ cutoff of 0.63, derived from an approximately 60% response rate to FOLFOX	RECIST 1.1 response of the biopsied lesion and all target lesions; progression‐free and overall survival	Prospective interim analysis (*n* = 42 of 107 established PDOs); positive predictive value 0.78, negative predictive value 0.80, AUROC 0.88 (target lesions); median PFS 10.1 vs. 2.4 months (*p* = 0.016); median OS 33 vs. 15 months (*p* = 0.049)
Pancreatic ductal adenocarcinoma (HOPE trial) [27]	Heterogeneous tissue sources: endoscopic ultrasound–guided fine‐needle biopsy, interventional‐radiology core biopsy, surgical resection, ascites/pleural fluid	16% of enrolled patients overall (13/76); 42% (13/31) among specimens yielding sufficient tumor cells	Median 96 days (range, 35–265) to initial drug‐sensitivity testing	Individualized, patient‐specific drug panel (not a fixed panel); included gemcitabine, 5‐FU, oxaliplatin, SN‐38, paclitaxel, and targeted agents	4 days	CytoTox‐Glo	Area under the curve (Simpson's rule)	Jenks natural‐breaks classification; a regimen was called “sensitive” if any one component drug scored AUC < 0.56	RECIST v1.1 disease control (partial response + stable disease) versus progressive disease	Pilot feasibility trial; retrospective correlation in 12 evaluable patients
Pancreatic ductal adenocarcinoma (neoadjuvant setting) [28]	Surgical resection and fine‐needle aspiration/biopsy	Surgical specimens: 71%–76%; FNA/FNB specimens: 53%–56%	Standard protocol: 60–90 days from tissue collection to drug‐sensitivity data. A separate exploratory rapid‐screening protocol, piloted on 6 samples, achieved 7–23 days (mean, 15)	Five standard‐of‐care drugs (gemcitabine, paclitaxel, SN‐38, 5‐FU, oxaliplatin), 9‐point dose–response curves	5 days	CellTiter‐Glo (ATP luminescence)	Normalized AUC; summed across regimen components (AUC_s_ᵤ_m_) for multidrug regimens	Tertile split by z‐score (top third = resistant, bottom third = sensitive, middle third = average)	Pathological response to neoadjuvant therapy (CAP grade); tumor cellularity; recurrence‐free survival	Retrospective correlation within a prospective cohort (117 enrolled; 21 in the neoadjuvant subgroup)
Breast cancer (including TNBC) [29]	Needle biopsy or surgical resection; primary and metastatic tumors	80.0% overall (60/75); 85.7% (48/56) in treatment‐naïve patients vs. 63.2% (12/19) in previously treated patients	Not reported	22‐agent panel (10 chemotherapeutic, 2 endocrine, 9 targeted, 1 traditional Chinese medicine); CellTiter‐Glo 3D, 384‐well format	6 days	CellTiter‐Glo 3D (ATP luminescence)	Half‐maximal inhibitory concentration (IC_50_) and dose–response curve slope	Not reported (qualitative sensitive/intermediate/resistant categorization; no numeric cutoff specified)	Case‐level comparison with documented treatment history and clinical response	Exploratory case series (individual illustrative patients); not a systematic cohort‐level correlation
Ovarian cancer (with endometrial and cervical cancer) [30]	Surgical resection	81.6% of enrolled patients generated organoids (31/38, across all gynecologic tumor types); 28 of 31 were expandable	Not reported	CellTiter‐Glo (Titer‐Glo) 3D assay, 96‐well format; drug testing performed on 10 of 15 established ovarian PDOs	7 days	ATP‐based luminescence	Area under the curve (IC_50_ also estimated)	Not reported	Clinical platinum‐sensitivity category (sensitive/resistant/refractory); CA‐125 trend; imaging	Retrospective, small case‐level correlation (4 illustrative patients); not statistically powered
Lung cancer (resected NSCLC/SCLC) [31]	Surgically resected lung tumor tissue	67% overall (53/79); 71% adenocarcinoma (35/49), 48% squamous (11/23), 100% SCLC and rare histologies	Organoids processed within 3 h of resection; “established” defined as survival beyond 3 passages and culture maintained ≥ 3 months (or expandable/cryopreservable within 3 months)	Genotype‐directed panel (EGFR TKIs erlotinib, afatinib, osimertinib, poziotinib; MEK inhibitor trametinib; SOS1 inhibitor BI‐3406; cisplatin + etoposide for SCLC)—not a uniform fixed panel	6 days (3‐day pre‐incubation, then 6 days of drug exposure with media/drug exchange every 3 days)	Cell Counting Kit‐8 (CCK‐8, formazan‐based viability)	Half‐maximal inhibitory concentration (IC_50_); sensitivity index (IC_50_ ÷ steady‐state trough drug concentration)	Not reported (no fixed cohort‐wide cutoff; comparisons made relative to matched cell lines)	Single illustrative patient: osimertinib response following organoid‐predicted EGFR‐exon‐20‐mutant sensitivity	Single‐case proof‐of‐concept correlation (partial response, radiographic and CEA decline); cohort‐level analysis focused on establishment predictors and association between long‐term culture (> 10 passages) and cancer‐specific survival
Renal cell carcinoma (air–liquid interface system) [32]	Surgically resected renal tumor tissue (clear cell RCC, papillary RCC, urothelial carcinoma, oncocytoma, chromophobe RCC)	72% overall culture success; 77% (20/26) for clear cell RCC specifically	Passaged after 21–30 days (1:3 split ratio); considered established once viable/passageable beyond 30 days in culture	Air–liquid interface (ALI), collagen‐based 3D culture; 10 organoids treated with cabozantinib (targeted TKI) or nivolumab (anti‐PD‐1)	1 week	Hematoxylin‐and‐eosin–based viable‐area quantification (Fiji image analysis)	Categorical response tiers with defined viability‐reduction bounds: no response, weak (≤ 1/3 reduction), medium (≤ 2/3 reduction), strong (> 2/3 reduction)	Numeric bounds embedded in the tier system (1/3 and 2/3 viability‐reduction cutoffs); no externally validated or clinically anchored threshold	None reported (no matched patient treatment outcome); nivolumab response correlated instead with CD8+ T‐cell density in the matched tissue (a biological, not clinical, correlate)	Proof‐of‐concept/exploratory only; authors explicitly note prospective clinical correlation is still needed
Gastric cancer [33]	Surgically resected tumor tissue	92.3% (12/13)	Not reported (drug testing initiated 4 days post‐seeding)	CellTiter‐Glo 2.0, 21‐drug panel, 384‐well format	72 h	ATP luminescence, dose–response curve	Area under the curve	Tertile split (sensitive/intermediate/resistant, 33%/34%/33%)	Retrospective clinical response (imaging, tumor markers)	Retrospective, single‐center; concordant in 6 of 8 evaluable cases (75%)

Modeling the TME introduces additional complexity. Standard PDO cultures are enriched for epithelial tumor cells and therefore lack many of the stromal and immune components that influence tumor progression in vivo. Recent work has begun to address this gap. Neal and colleagues demonstrated that tumor‐infiltrating lymphocytes can be maintained and functionally assessed within organoid platforms [34]. Other studies incorporating cancer‐associated fibroblasts have provided further insight into stromal signaling interactions [35].

Even so, organoids do not fully replicate vascular networks, systemic immune regulation, endocrine influences, or mechanical forces present within living tissues. In practice, they occupy a position between reductionist cell culture and in vivo models. They simplify the surrounding ecosystem while preserving key tumor‐intrinsic features. That balance between fidelity and controllability largely explains their growing relevance in translational cancer research [18, 21, 36].

### 4.2 Genetic and Epigenetic Fidelity Compared to Primary Tumors

The credibility of any disease model rests on its molecular fidelity. In the case of PDOs, evidence supporting genomic concordance with parental tumors is substantial. Whole‐exome sequencing and copy number analyses repeatedly demonstrate preservation of driver mutations and major oncogenic pathways across serial passages [17, 19, 22, 23, 37]. CRC organoid biobanks, in particular, have confirmed maintenance of patient‐specific mutational profiles and chromosomal instability patterns [17].

Beyond genomic alterations, transcriptional identity is largely conserved. Transcriptomic studies indicate that organoids retain lineage‐associated gene expression programs and tumor subtype characteristics [19, 22, 23]. Emerging epigenomic data suggest that DNA methylation patterns and chromatin accessibility states are also preserved during early and intermediate passages [23, 37]. While prolonged culture may introduce some epigenetic divergence, the degree of molecular stability observed in controlled systems supports their use in mechanistic studies. This molecular continuity is not only reassuring from a descriptive standpoint. It enables functional experimentation. Because organoids retain the genetic and transcriptional framework of the original tumor, they allow researchers to examine how those alterations manifest biologically. When genomic profiling is combined with drug response testing, organoids provide a means of connecting molecular alterations with therapeutic outcomes [21, 36, 38]. This integration moves beyond mutation cataloging and toward functional interpretation.

### 4.3 Advantages of PDOs Compared With Traditional 2D Cultures and Patient‐Derived Xenografts (PDXs)

2D cancer cell lines have contributed enormously to our understanding of cancer biology. At the same time, prolonged growth under monolayer conditions can result in clonal selection and genetic drift, sometimes leading to models that diverge from the tumors they were meant to represent [18, 21]. Organoids reduce some of these limitations by maintaining three‐dimensional structure, differentiation hierarchies, and aspects of tumor heterogeneity [17, 19, 22]. Although selection pressures are not entirely eliminated, organoids tend to retain closer molecular and phenotypic resemblance to patient tissue than long‐established cell lines.

When considered alongside PDX models, PDOs offer distinct advantages. Xenografts preserve tumor growth within a living organism and maintain vascular interactions, but they require considerable time and resources and are not easily adapted for rapid drug screening [21, 36]. Organoids, by contrast, can often be established within weeks and expanded for parallel testing of multiple therapeutic agents [23, 38]. Co‐clinical investigations demonstrating correlation between organoid drug responses and patient outcomes further reinforce their translational relevance [3, 39]. For these reasons, PDOs are often viewed as a practical bridge. They provide greater biological fidelity than conventional cell lines while remaining more experimentally manageable than animal models.

### 4.4 Limitations: Loss of Stromal and Immune Components and Batch Variability

Despite their strengths, PDO systems are not without limitations. Standard cultures do not fully reproduce vascular networks, systemic immune regulation, or the biomechanical context present within tumors in vivo [18, 21, 36]. Immune‐organoid co‐culture approaches represent meaningful progress, yet they remain technically demanding and lack uniform standardization.

Matrix dependence also introduces variability. Most organoid systems rely on biologically derived extracellular matrices such as Matrigel, which can vary between batches and influence cellular behavior [36]. The development of synthetic or chemically defined matrices is ongoing and may improve reproducibility across laboratories.

Finally, establishment efficiency differs among tumor types, and in vitro growth conditions may favor expansion of certain subclones [17, 24]. This potential for selective representation underscores the need for validation when translating organoid findings into broader biological conclusions. Taken together, these considerations do not diminish the value of PDOs. Rather, they define the context in which organoid data should be interpreted. While not complete replicas of tumors in vivo, PDOs remain among the most biologically informative and experimentally adaptable human cancer models currently available.

## 5 PDOs in Personalized Drug Testing

PDOs have emerged as a central functional platform for evaluating individualized therapeutic responses in oncology. In contrast to genomics‐only precision approaches, PDO‐based pharmaco‐typing enables direct assessment of tumor viability, growth dynamics, and pathway activation in response to chemotherapeutic and targeted agents, thereby providing functional validation of treatment hypotheses [40].

### 5.1 Methodological Principles of PDO‐Based Drug Testing

Translating PDO drug‐sensitivity data into a clinically actionable readout requires a defined pipeline: tissue acquisition, organoid establishment, drug exposure, viability readout, and comparison against a clinical outcome. Table 1a summarizes this pipeline across the major cancer types reviewed here, and several patterns emerge that bear directly on reproducibility and translational readiness.

Establishment rates cluster around 50%–90% for surgically resected tissue (67% in lung, 80% in breast, 92% in gastric cancer, and 52%–90% in colorectal and gynecologic series), but drop sharply for needle‐biopsy or fine‐needle aspiration specimens; 16% of enrolled pancreatic cancer patients overall via endoscopic or percutaneous biopsy, versus 71%–76% from matched surgical tissue in the same disease [27, 28, 29, 30, 31, 33, 41]. Histologic subtype also matters within a single tissue source: in lung cancer, adenocarcinoma established at 71% versus 48% for squamous cell carcinoma from the same resected‐tissue pipeline [31]. Prior treatment exposure similarly lowers establishment success: in breast cancer, PDOs from previously treated patients established at 63.2%, compared with 85.7% in treatment‐naïve patients [29].

Turnaround time varies more than is generally acknowledged in the literature, and reported figures require careful reading. The colorectal OPTIC trial reports a median of 61 days to a biobank‐ready culture plus roughly two additional weeks for screening [25]; the pancreatic neoadjuvant study's standard protocol requires 60–90 days, while a separate small‐scale rapid‐screening pilot (6 samples) demonstrated that 7–23 days is achievable at low passage number [28]. The lung and renal cell carcinoma studies define “establishment” itself by a culture‐longevity criterion rather than a fixed calendar target, survival beyond 3 passages and maintenance for at least 3 months in lung cancer, and viability beyond 30 days with a 21–30‐day passaging interval in renal cell carcinoma [31, 32]. Distinguishing standard from exploratory rapid‐screening timelines, and distinguishing a longevity‐based establishment definition from a fixed turnaround target, is important since this materially affects how “clinically actionable” a given protocol is in practice.

Assay platforms have converged on ATP‐luminescence or nuclear‐fluorescence viability readouts, most paired with dose–response curves and AUC‐type or IC_50_‐type summary metrics rather than single‐concentration endpoints. CyQUANT and CellTiter‐Glo performed comparably where directly compared (CRC), suggesting that choice of readout platform is less consequential than choice of response metric or threshold. The renal cell carcinoma air–liquid‐interface platform is the exception, using semi‐quantitative histologic scoring rather than a viability assay, reflecting its immune‐competent culture format [26, 32].

Predefined response thresholds are more common in this literature than is often assumed, though almost none are externally validated. The colorectal, pancreatic, and gastric cancer studies each convert continuous AUC or GR_a_ᴜᴄ values into a binary or tertile sensitive/resistant call, via a Jenks natural‐breaks split, a z‐score tertile split, or a cutoff back‐calculated from a population response rate (e.g., 0.63 for CRC, derived from an approximately 60% FOLFOX response rate) [25, 26, 27, 28, 33]. The renal cell carcinoma platform similarly embeds numeric bounds into its categorical response tiers (weak, medium, and strong response defined by 1/3 and 2/3 viability‐reduction cutoffs), though these were not derived from or validated against patient outcomes [32]. These are internally consistent classification schemes rather than independently derived or externally validated cutoffs, and each is specific to its own drug panel and patient population; a “sensitive” call under one study's threshold is not interchangeable with another's. The breast, ovarian, and lung cancer studies summarized here report no fixed cohort‐wide numeric threshold, relying instead on qualitative categorization or case‐level comparison [29, 30, 31]. This inconsistency, rather than a complete absence of thresholds, is the more accurate characterization of the field's current reproducibility gap.

Validation status ranges from single‐arm exploratory case series to prospectively interim‐validated cohorts. The colorectal OPTIC trial is the most mature entry in this table, testing a pre‐specified cutoff prospectively against RECIST response, progression‐free survival, and overall survival in an interim analysis of 42 patients [25]. The pancreatic and gastric cancer studies offer retrospective correlative evidence in cohorts of 12–21 evaluable patients [27, 28, 33]. The renal cell carcinoma platform has not yet been compared with any matched patient outcome at all, relying instead on an internal biological correlate (nivolumab response tracking with CD8+ T‐cell density in the matched tissue) [32]. The breast, ovarian, and lung cancer evidence summarized here is limited to illustrative individual case comparisons rather than cohort‐level statistics, which should be reflected in how strongly these findings are weighted in the surrounding text [29, 30, 31].

A subset of studies test a single mechanistic hypothesis, targeted‐agent sensitivity or immune‐mediated cytotoxicity, rather than a full drug‐screening pipeline, and are summarized separately in Table 1b for comparability.

**Table 1b:** Supplementary—single‐mechanism extension studies (not standard drug‐panel screening).

Cancer type	Sample source	Study type	Agent/mechanism tested	Readout	Key finding	Reference
Triple‐negative breast cancer	Patient‐derived organoids and matched patient‐derived xenograft organoids	Single‐agent targeted therapy	WEE1 inhibitor AZD1775	DNA damage markers; G2/M cell‐cycle arrest; concordance with documented clinical response	Concordant response across organoids, xenograft organoids, and clinical outcome	[42]
Renal cell carcinoma	Organoid–immune cell co‐culture	Immune co‐culture functional assay	Antigen‐specific CAR‐T cell activity	Cytokine release; apoptosis induction in tumor organoids	Increased cytokine release and apoptosis relative to control co‐cultures	[43]

### 5.2 Case Studies Across Cancer Types

#### 5.2.1 CRC

In mCRC, Smabers et al. [26] systematically evaluated methodological factors influencing PDO drug screening performance and assessed the capacity of optimized organoid assays to correlate with patient responses to standard chemotherapeutic regimens, including fluoropyrimidine‐, irinotecan‐, and oxaliplatin‐based treatments. The study demonstrates that assay design and data analysis parameters significantly affect the predictive performance of PDO‐based pharmaco‐typing in clinical settings.

CRC PDO platforms have also demonstrated preservation of histopathological architecture and molecular characteristics of parental tumors, supporting their relevance as translational drug‐testing models. In addition to chemotherapy prediction, CRC PDOs have been used to examine targeted therapy responses. In a study evaluating FGFR4‐variant and wild‐type CRC organoids, treatment with FGFR4 inhibitors (FGFR4‐IN‐1 and erdafitinib) reduced organoid growth and decreased phosphorylation of downstream signaling mediators, including ERK1/2, AKT, and STAT3 [44]. The authors report that targeted inhibition affected both FGFR4‐variant and wild‐type organoids, and further studies are required to determine genotype‐specific selectivity.

These findings collectively demonstrate the utility of CRC PDOs for both methodological optimization of chemotherapy prediction and functional interrogation of pathway‐directed therapies.

#### 5.2.2 PDAC

PDAC serves as a clinically advanced example of prospective PDO integration. In a feasibility study (HOPE trial), Grossman et al. [27] generated PDAC organoids from biopsy specimens and conducted drug‐sensitivity testing, comparing organoid drug response profiles with patient clinical outcomes. Drug testing was feasible in a subset of enrolled patients, and PDO drug response profiles were associated with disease control as determined by radiographic evaluation.

In another study, organoids derived from PDAC surgical specimens were used to evaluate chemotherapy sensitivity in the neoadjuvant therapy (NAT) setting [28]. The study reported that pathological response following NAT correlated with PDO chemotherapy response, and drug testing could be performed within a clinically relevant timeframe after tumor resection. However, an important distinction in interpreting these findings is whether organoid–patient concordance represents retrospective validation or genuine prospective prediction. Among 21 patients enrolled in the neoadjuvant cohort, only four patients (20%) had a pre‐treatment biopsy submitted for PDO establishment, although three of these four samples (75%) successfully generated organoids. In contrast, after NAT, 18 patients underwent surgical resection and PDOs were successfully established from 11 specimens (61%) [28]. Thus, although pre‐treatment biopsies demonstrated a numerically higher establishment rate, their limited availability substantially restricts the number of patients for whom PDOs could theoretically inform treatment selection before therapy begins. Importantly, the post‐treatment surgical PDOs provide valuable retrospective information regarding the biological consequences of NAT, but they cannot prospectively predict the response to the treatment that has already been administered.

This distinction is critical for clinical translation. Pre‐treatment PDOs have the greatest theoretical value because they can be generated before exposure to NAT and could, in principle, be used to compare alternative regimens and identify patients unlikely to benefit from standard treatment. However, the existing evidence remains insufficient to establish that pre‐treatment PDO drug sensitivity can reliably forecast pathological response at the individual‐patient level. In the PDAC neoadjuvant study, the association between PDO chemotherapy sensitivity and pathological response is encouraging, but the pre‐treatment biopsy subgroup consisted of only four patients, making this finding hypothesis‐generating rather than definitive [28]. Consequently, retrospective correlations from post‐treatment surgical specimens should not be equated with prospective predictive validity. Future studies should prioritize larger, multicenter cohorts in which PDOs are generated from treatment‐naïve diagnostic biopsies, drug‐response testing is completed before NAT, and predefined PDO response thresholds are prospectively compared with pathological response, recurrence‐free survival, and other patient‐centered outcomes. Until such validation is available, pre‐treatment PDOs should be regarded as promising predictive biomarkers rather than established tools for directing neoadjuvant treatment.

#### 5.2.3 Breast Cancer

In triple‐negative breast cancer (TNBC), PDOs and PDX models were used to evaluate sensitivity to the WEE1 inhibitor AZD1775. The study reported concordant drug response patterns between matched PDOs and PDX tumors, and these ex vivo findings were compared with documented clinical treatment responses [42]. Mechanistically, AZD1775 treatment increased DNA damage markers and induced G2/M cell‐cycle arrest in TNBC organoids, supporting WEE1 inhibition as a functionally validated therapeutic vulnerability.

Beyond TNBC, Huizi Wu et al. [29] established 60 organoids from 75 breast cancer samples, achieving an 80.0% establishment success rate. Drug screening was performed using a panel of 22 agents, and the study found consistency between PDO drug susceptibility results and matched patient clinical responses. These findings support the feasibility of PDO‐based drug testing in breast cancer, including cases with prior therapy exposure.

Additionally, biomaterial‐based platforms have been investigated to enhance ex vivo drug testing. Tunable hydrogel systems support the growth of patient‐derived breast cancer organoids and influence measured drug sensitivity, indicating that ECM characteristics can affect pharmacologic response in 3D culture models [45].

#### 5.2.4 Ovarian Cancer

In ovarian cancer, Chen et al. [30] established PDOs and performed in vitro drug‐sensitivity testing, reporting that organoid responses corresponded with clinical platinum‐sensitivity status in evaluated cases, including platinum‐sensitive, platinum‐resistant, and refractory categories. Similarly, Chang et al. [46] reported that ovarian cancer PDOs preserved the mutational profiles and copy number alterations of their parental tumors and exhibited differential responses to chemotherapeutic agents in vitro. These results indicate that ovarian PDOs retain the genomic characteristics of primary tumors and are suitable for functional drug response assessment.

#### 5.2.5 Lung Cancer

In surgically resected lung cancer, PDOs were used for biomarker and drug testing, preserving histologic and genetic features of parental tumors and demonstrating genotype‐aligned therapeutic sensitivity (e.g., EGFR mutation‐associated response to osimertinib) [31].

In non–small cell lung cancer (NSCLC), organoids identified overexpression of AKR1B10 in chemoresistant models, and repurposing of epalrestat restored chemotherapy sensitivity in organoid and xenograft systems [47].

#### 5.2.6 RCC

RCC PDOs have been successfully established using both matrix‐embedded and air–liquid interface (ALI) culture systems, demonstrating preservation of histopathological architecture and tumor‐specific molecular features relative to their parental tumors [32, 43]. In the ALI platform, organoids retained stromal and immune components, including CD8^+^ T cells, and showed close transcriptomic similarity to matched tumor tissue. Functionally, RCC PDOs have been utilized for targeted drug screening, revealing heterogeneous responses to agents such as cabozantinib. Immune‐based evaluations have also been conducted: ALI PDOs were tested with nivolumab in vitro, demonstrating variable responses across samples. In parallel, separate RCC organoid systems were used to assess antigen‐specific CAR‐T cell activity in co‐culture experiments, demonstrating immune‐mediated cytotoxicity against tumor organoids [43].

#### 5.2.7 GC

GC PDOs were generated from surgical specimens and shown to retain histologic features and genetic alterations consistent with their matched primary tumors [33]. Drug susceptibility testing was performed using standard‐of‐care chemotherapeutic agents, and the study reports concordance between PDO drug response profiles and corresponding patient clinical outcomes in a subset of evaluated cases.

### 5.3 PDOs as Predictors of Chemotherapy and Targeted Therapy Response

Across tumor types, the predictive value of PDOs has been demonstrated in platinum‐based chemotherapy in ovarian cancer, neoadjuvant chemotherapy in PDAC, Fluoropyrimidine‐, irinotecan‐, and oxaliplatin‐based regimens in mCRC, WEE1 inhibition in TNBC, FGFR4 inhibition in CRC, EGFR‐targeted therapy in lung cancer [26, 28, 30, 31, 42, 44]. Collectively, these studies demonstrate that PDOs can serve as predictors of conventional chemotherapy response and as functional validators of targeted therapy vulnerabilities, including drug repurposing strategies [47].

### 5.4 Tumor‐Type‐Specific Determinants of Organoid Establishment

Establishment efficiency and media requirements differ substantially between epithelial and non‐epithelial malignancies, and the standard matrix‐embedded protocol does not transfer to several tumour types. Glioblastoma models are generally propagated as neurosphere‐derived or cerebral‐organoid‐based systems rather than matrix‐embedded epithelial cultures, depend on neural rather than Wnt‐driven media, and lose the hypoxic and perivascular niches that drive treatment resistance [41]. Prostate cancer organoids have historically shown low long‐term establishment rates from primary tissue, with androgen‐receptor‐positive luminal cultures frequently overgrown by benign basal epithelium, and require androgen supplementation and additional pathway inhibition to be sustained [48]. Hematological malignancies have no epithelial compartment and cannot be modelled by conventional organoid protocols at all; available systems instead reconstruct a marrow or lymphoid niche using stromal scaffolds and co‐culture, which limits their comparability with solid‐tumor PDO data [49]. These differences mean that the establishment rates, turnaround times and response thresholds reported in Table 1a cannot be extrapolated across tumor types, and they are a substantial part of the reason the evidence base summarized in Figure 1 remains confined to solid epithelial tumors.

**Figure 1:**
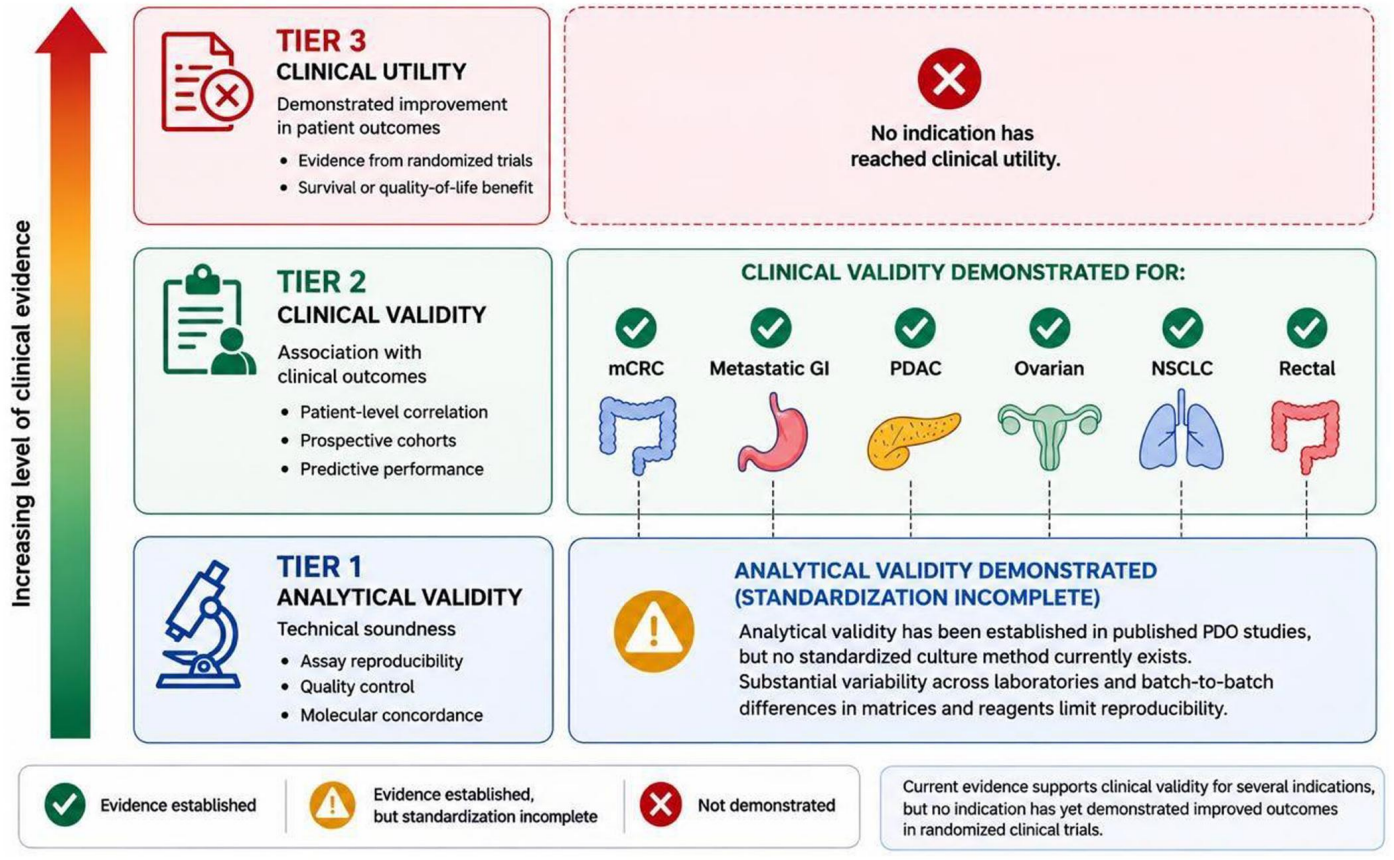
Evidence‐grade framework for clinical validation of PDOs across selected solid tumors. Tumor indications are shown at the highest level of evidence currently supported by published studies. Tier 1 represents analytical validity, Tier 2 represents clinical validity, and Tier 3 represents clinical utility. Only tumor types with sufficient published prospective or co‐clinical patient–outcome concordance data to permit evidence grading are included in this framework; additional clinical studies and tumor types are summarized in Table 1a. No tumor indication has yet achieved Tier 3 clinical utility.

### 5.5 Incorporation Into Co‐Clinical and N‐of‐1 Trials

The integration of PDOs into co‐clinical and personalized trial designs is actively progressing. The HOPE PDAC feasibility trial represents a prospective co‐clinical model correlating PDO drug sensitivity with clinical outcomes [27]. The OPTIC CRC study framework integrates PDO pharmaco‐typing within a prospective clinical trial infrastructure [26]. Biopsy‐specific PDO establishment success and drug‐testing timelines highlight key practical considerations for clinical translation (Table 1c).

Breast cancer PDO biobanking efforts demonstrate applicability in advanced and recurrent disease, supporting potential N‐of‐1 decision‐making frameworks [29]. These studies indicate that PDO pharmaco‐typing is moving from retrospective and observational correlation toward prospective clinical evaluation; however, prospective feasibility should not be equated with demonstrated clinical utility, and randomized interventional studies remain necessary to determine whether PDO‐guided treatment selection improves patient outcomes (Table 2).

## 6 Integration of Patient‐Derived Organoids With Precision Oncology Platforms

Precision oncology increasingly requires systems that connect molecular stratification to functional therapeutic testing in models that retain patient‐specific tumor biology. PDOs preserve key histologic and genomic features of the source tumor and can be scaled for drug testing, making them attractive platforms for translational precision medicine. Their impact is amplified when integrated with next‐generation sequencing (NGS), CRISPR‐based functional genomics, immune co‐culture systems, and microfluidic organ‐on‐chip approaches.

### 6.1 PDOs + NGS for Patient Stratification and Genotype–Phenotype Linkage

NGS can identify actionable alterations and genomic features (e.g., broad mutation landscapes, structural variation), but therapeutic response is not reliably inferred from genotype alone in many tumors. Lung cancer organoids characterized by whole‐genome sequencing (WGS) provide an example of how comprehensive genomic profiling can be paired with organoid establishment to support downstream functional interrogation and therapy development [50]. More broadly, clinical co‐trial organoid biobanks have shown that PDO drug sensitivity patterns can align with patient outcomes in metastatic gastrointestinal cancers, supporting the concept of phenotype‐first validation in a patient‐matched system [3].

### 6.2 PDOs + CRISPR/Cas9 for Functional Genomics

CRISPR perturbation in PDOs enables causal identification of genetic dependencies, drug resistance mechanisms, and synthetic lethal interactions within a 3D patient‐derived context. A comprehensive review of PDO–CRISPR integration summarizes how CRISPR knockout/CRISPRi/CRISPRa approaches can be layered onto PDO workflows for target discovery and precision therapeutic hypothesis testing [51]. In CRC, CRISPR editing in tumor organoids has been used to functionally validate candidate drivers in organoid‐based systems, illustrating feasibility of gene‐function interrogation in 3D tumor models. In pancreatic cancer models, integrated genome editing and screening approaches have been used to connect genetic perturbations to drug response phenotypes in organoid‐like systems, supporting drug–gene interaction mapping relevant to therapy optimization [52].

However, interpretation of PDO–CRISPR experiments requires particular caution because unintended editing events can introduce phenotypes that are incorrectly attributed to the intended genetic perturbation. Off‐target cleavage, large genomic rearrangements, copy‐number alterations, or selection of pre‐existing clones may alter cellular fitness and drug sensitivity, potentially producing false mechanistic conclusions. These concerns are especially relevant in cancer PDOs, in which genomic instability and clonal heterogeneity are intrinsic features of the disease. Therefore, the feasibility of CRISPR‐based perturbation in PDOs should be distinguished from the specificity and biological validity of the resulting phenotypes.

Accordingly, target specificity should be experimentally validated rather than inferred solely from guide‐RNA design or the presence of the expected on‐target mutation. Recommended approaches include sequencing of predicted off‐target loci, comparison with appropriate parental or non‐edited controls, and, where feasible, broader genomic assessment such as WGS. The use of high‐fidelity Cas9 variants or alternative editing systems may further reduce unintended cleavage. Evidence from organoid genome‐editing studies demonstrates that WGS can be used to assess genome‐wide off‐target effects, while high‐fidelity Cas9 systems have been developed specifically to improve editing specificity. However, these safeguards have not been uniformly applied across PDO–CRISPR studies, and the current literature does not support assuming that all reported PDO–CRISPR phenotypes have undergone comprehensive genome‐wide specificity validation.

Therefore, future PDO–CRISPR studies should incorporate predefined quality‐control criteria, including confirmation of the intended edit, assessment of predicted off‐target sites, appropriate isogenic or parental controls, and, particularly for mechanistic studies in which a phenotype will be directly attributed to a specific gene, genome‐wide sequencing when feasible. These measures are important not only for experimental reproducibility but also for excluding genomic instability or clonal selection as alternative explanations for observed drug‐resistance phenotypes. Recent large‐scale PDO resources combining whole‐genome characterization with genome‐wide CRISPR screening further illustrate the value of integrating genomic quality control with functional perturbation studies. Taken together, PDO–CRISPR platforms provide a powerful framework for functional genomics and drug‐resistance research, but conclusions regarding gene‐specific mechanisms should be interpreted in proportion to the extent of editing‐specificity and genomic validation performed.

### 6.3 Immuno‐Organoids: PDOs + Immune Co‐Cultures for Immunotherapy Testing

Because classic epithelial PDOs lack immune components, immune co‐culture (immuno‐organoid) platforms aim to model tumor–immune interactions and evaluate immunotherapy strategies ex vivo. A focused review in Cancer Research outlines the rationale and approaches for combining tumor organoids with immune elements to support preclinical testing for precision cancer immunotherapy [5]. Another review summarizes organoid‐based strategies for modeling the tumor immune microenvironment and testing Immunotherapeutics, emphasizing methodological options for immune incorporation and response readouts [53].

### 6.4 PDOs + Organ‐on‐Chip and Microfluidics for Dynamic Tumor–Immune–Drug Interactions

Microfluidic organ‐on‐chip systems address static culture limitations by enabling perfusion, controlled transport, multi‐compartment co‐culture, and time‐resolved exposure, which are especially relevant for tumors shaped by stromal barriers and immune exclusion. A patient‐derived pancreatic cancer organ‐on‐chip platform integrating PDOs with key TME components (including stromal and immune elements) demonstrates how microfluidics can enhance ex vivo modeling of drug response under more physiologic condition [54]. Foundationally, organoids‐on‐a‐chip has been positioned as a route to improve organoid maturity and experimental control through micro‐engineered microenvironments [55]. Tumor‐on‐chip platforms with perfused microvascular networks have also shown feasibility for culturing patient‐derived tumor organoids and studying drug responses under vascularized transport conditions [56]. In parallel, lung cancer organ‐chip models have been used to recapitulate organ microenvironment–dependent cancer behaviors and responses to targeted therapy, illustrating the broader utility of organ‐chip cancer modeling [57]. At the systems level, integrated multi‐organoid “body‐on‐a‐chip” approaches have been used for multi‐tissue drug screening and toxicity evaluation, supporting future directions toward systemic therapy modeling [58].

## 7 ⁠Clinical Translation and Ongoing Trials

The clinical translation of PDOs has advanced considerably over the past decade; however, their role in routine clinical practice remains under active investigation. A central question has been whether drug responses observed in PDOs reliably predict treatment outcomes in patients. Although numerous studies have demonstrated encouraging associations between organoid drug sensitivity and clinical outcomes, the strength of evidence varies according to the endpoint evaluated. Current evidence supports several distinct levels of validation, including concordance with radiographic response, correlation with progression‐free survival (PFS), prediction of therapeutic resistance, prospective feasibility of PDO‐guided testing, and, to a much lesser extent, demonstration of clinical utility through treatment decision‐making.

Unlike genomic profiling, which identifies potential therapeutic targets based on molecular alterations, PDOs provide a functional assessment of tumor response that incorporates the combined effects of tumor heterogeneity, signaling interactions, and adaptive resistance mechanisms [3, 38]. Nevertheless, although functional testing complements genomic profiling, its clinical utility has not yet been definitively established and requires further prospective validation.

### 7.1 Clinical Validation in mCRC

Metastatic colorectal cancer (mCRC) currently provides the strongest clinical evidence supporting PDO translation. Several prospective observational studies have demonstrated concordance between organoid drug sensitivity and radiographic treatment response. Ooft et al. [39] reported that responses of CRC PDOs to irinotecan‐ and oxaliplatin‐based chemotherapy were associated with clinical treatment responses, supporting the feasibility of functional drug testing in this setting. Similarly, Vlachogiannis et al. [3] demonstrated concordance between PDO drug responses and clinical outcomes in metastatic gastrointestinal cancers, including the accurate identification of both sensitive and resistant tumors. Evidence has also begun to extend beyond radiographic response. Smabers et al. [25] demonstrated that PDO drug sensitivity scores were associated with PFS in patients with mCRC, suggesting that PDOs may serve as functional predictive biomarkers. Although these findings are encouraging, the available studies remain predominantly observational, and additional prospective validation in larger multicenter cohorts is required before routine clinical implementation.

### 7.2 Pancreatic Cancer and Other Solid Tumors

Evidence supporting PDO‐guided prediction has also accumulated in pancreatic, ovarian, breast, lung, and rectal cancers, although the degree of clinical validation varies considerably among tumor types. In pancreatic ductal adenocarcinoma, Tiriac et al. [23] demonstrated that PDO drug responses correlated with chemotherapy sensitivity observed in patients, supporting the feasibility of individualized therapeutic testing. Similar associations have been reported in ovarian cancer, where de Witte et al. [59] observed concordance between organoid responses and clinical responses to platinum‐based chemotherapy. In lung cancer, Kim et al. [60] showed that organoid responses to EGFR‐targeted therapies were consistent with both tumor genotype and clinical outcomes. Ganesh et al. [61] further demonstrated the feasibility of predicting chemoradiotherapy responses using rectal cancer organoids. Collectively, these studies suggest that PDOs can reproduce clinically relevant patterns of therapeutic sensitivity and resistance across multiple tumor types. However, most investigations involve relatively small cohorts and are designed to evaluate predictive concordance rather than demonstrate improved patient outcomes following PDO‐guided treatment selection. Consequently, the level of evidence remains heterogeneous across different malignancies.

### 7.3 Biobanking and Standardization

The transformation from promising laboratory findings to replicable translational platforms was mainly reliant on biobanking infrastructure. Clevers described the creation of organoid culture systems capable of long‐term growth while retaining critical epithelial properties [62]. Building on this foundation, Driehuis et al. [11] released detailed techniques for creating patient‐derived cancer organoids appropriate for drug testing. These methodological breakthroughs focused on repeatability, culture conditions, and quality control. The development of living organoid biobanks, such as the breast cancer organoid repository described by Sachs et al. [19], has allowed for the systematic analysis of tumor heterogeneity and therapeutic response in large patient cohorts. Long‐term cryopreservation and re‐expansion enable repeated testing and comparative evaluation of therapeutic options, which is critical for translational scalability.

However, the clinical relevance of PDO biobanking depends not only on the ability to preserve and expand organoids but also on establishment success and whether organoids can be generated and screened within the therapeutic decision window. Quantitative data demonstrate substantial variation according to tissue source. In PDAC, surgical specimens achieved establishment rates of approximately 71%–76%, whereas FNA/FNB specimens achieved approximately 53%–56% in the neoadjuvant study [28]. In the broader HOPE cohort, PDOs were successfully generated from only 16% of enrolled patients overall, although the denominator included patients from several tissue‐acquisition pathways [27]. In mCRC, the OPTIC study reported an overall establishment rate of 52% (107/205), with improvement from 22% to 75% over the study period; however, the median time to a biobank‐ready culture was 61 days, followed by approximately two additional weeks for drug screening [25]. These findings demonstrate that establishment success and turnaround time are inseparable determinants of the clinical feasibility of PDO‐based testing.

Selection bias represents an additional concern when interpreting establishment rates. Surgical resections generally provide substantially more tissue and may therefore yield higher establishment success, but patients undergoing surgery represent a selected population with potentially different disease characteristics, including greater resectability and, in some settings, better performance status, than patients with unresectable or metastatic disease. Conversely, core biopsies are more representative of the specimens available at the time treatment decisions are made, but their smaller volume and potentially lower tumor cellularity may reduce establishment efficiency. The PDAC neoadjuvant cohort illustrates this distinction particularly clearly: pre‐treatment biopsies were available for only 20% of patients in the neoadjuvant cohort, although 75% of these biopsy‐derived cultures were successfully established, whereas 61% of post‐NAT surgical specimens generated PDOs [28]. Thus, the apparently higher establishment rate of pre‐treatment biopsies should not be interpreted as evidence of broad clinical feasibility because the biopsy subgroup was very small. More broadly, reliance on surgical specimens in PDO studies may lead to overestimation of establishment success and underrepresentation of patients for whom treatment‐guiding PDOs would be most clinically relevant.

Importantly, reported timelines vary considerably between studies and should be interpreted according to the complete workflow rather than the duration of the final drug‐exposure assay alone. Standard PDAC neoadjuvant protocols required approximately 60–90 days from tissue collection to drug‐sensitivity data, whereas an exploratory rapid‐screening protocol achieved testing within 7–23 days in only six samples [28]. The colorectal OPTIC study required a median of 61 days to obtain a biobank‐ready culture, followed by approximately two additional weeks for screening [25]. Therefore, rapid protocols should be distinguished from validated routine workflows, and short drug‐exposure periods should not be conflated with short overall turnaround times. A clinically useful PDO workflow ultimately requires reproducible tissue acquisition, establishment, expansion, quality control, and drug‐testing timelines that fit within the therapeutic decision window rather than merely demonstrating that rapid testing is technically possible in selected samples (see Table 1c).

**Table 1c:** Biopsy‐specific PDO establishment and drug‐testing timelines relevant to clinical translation.

Cancer type/study	Tissue source	Establishment success	Time to drug‐sensitivity data	Clinical relevance/limitation	Reference(s)
PDAC, neoadjuvant cohort	Pre‐treatment FNA/FNB biopsy	75% (3/4); pre‐treatment biopsy available in only 20% (4/21) of neoadjuvant patients	Standard workflow: 60–90 days; exploratory rapid workflow: 7–23 days in 6 samples	Pre‐treatment PDOs offer the greatest potential for prospective prediction, but the sample size was very small; pathological‐response correlation therefore remains exploratory	[28]
PDAC, post‐NAT surgical specimens	Surgical resection after neoadjuvant therapy	61% (11/18)	60–90 days in standard workflow	Provides retrospective correlation with pathological response but cannot prospectively guide the neoadjuvant treatment already received	[28]
mCRC, OPTIC	18‐gauge metastatic‐lesion biopsy	52% overall (107/205); improved from 22% to 75% over the study period	Median 61 days to biobank‐ready culture + approximately 2 weeks for screening	Most mature prospective validation framework, but turnaround remains longer than many treatment‐decision windows	[25]
Breast cancer	Needle biopsy or surgical resection	80.0% overall; 85.7% treatment‐naïve vs. 63.2% previously treated	Not reported	Demonstrates establishment feasibility but does not provide a standardized biopsy‐to‐result clinical timeline	[29]
Lung cancer	Surgical resection	67% overall; 71% adenocarcinoma vs. 48% squamous carcinoma	Organoids processed within 3 h; establishment defined by survival beyond 3 passages and maintenance for ≥ 3 months	Establishment definition is longevity‐based rather than a fixed clinical turnaround target	[31]
Gastric cancer	Surgical resection	92.3% (12/13)	Drug testing initiated 4 days after seeding; assay duration 72 h	High establishment and short assay period, but evidence remains retrospective and single‐center	[33]

**Table 2:** Ongoing and translational clinical studies utilizing patient‐derived organoids for precision oncology.

Trial/study	Cancer type	Study design/goal	Key PDO application	Reference(s)
HOPE Trial	Pancreatic ductal adenocarcinoma	Prospective feasibility trial generating PDOs from biopsies	PDO drug sensitivity testing compared with patient clinical outcomes	[27]
OPTIC Study Framework	Colorectal cancer	Prospective clinical study integrating PDO pharmaco‐typing	Integration of PDO drug‐response data into clinical trial infrastructure	[25]
Ooft et al. clinical correlation study	Metastatic colorectal cancer	Prospective translational study	PDO chemotherapy sensitivity correlated with radiologic treatment response	[39]
Vlachogiannis et al. co‐clinical organoid study	Metastatic gastrointestinal cancers	Organoid–patient outcome comparison	High concordance between PDO drug responses and patient treatment outcomes	[3]
Smabers et al. translational cohort study	Metastatic colorectal cancer	Large cohort evaluation of PDO predictive performance	PDO drug‐sensitivity scores correlated with progression‐free survival	[26]

*Note:* Representative clinical trials and translational studies integrating PDO platforms into clinical workflows. The table highlights the cancer type, study design, and the role of PDOs in predicting treatment response, guiding therapy selection, or validating drug sensitivity profiles.

These findings further emphasize the need for standardization of culture conditions, ECM composition, medication exposure timing, assay endpoints, and quality‐control procedures before PDO testing can be broadly incorporated into clinical workflows. Establishment success should also be reported according to tissue source, tumor subtype, prior treatment exposure, and the proportion of enrolled patients from whom PDOs could ultimately be generated. Such reporting would allow more accurate assessment of the real‐world accessibility and scalability of PDO‐based precision oncology.

### 7.4 Regulatory and Ethical Considerations

Clinical implementation of PDO‐based drug testing requires demonstration of analytical validity, clinical validity, and clinical utility. Analytical validity has improved substantially through standardized culture protocols and quality‐control procedures [11].

Clinical validity is supported by prospective observational studies demonstrating associations between PDO drug responses and clinical outcomes in colorectal, pancreatic, gastrointestinal, ovarian, and other cancers.

Clinical utility, however, represents the highest level of evidence and requires demonstration that PDO‐guided therapeutic decisions improve patient outcomes compared with standard clinical management. At present, this evidence remains limited, and randomized prospective interventional studies will be required before routine clinical adoption can be recommended [38]. Figure 1 summarizes this three‐tier evidence landscape across the six cancer types with published PDO validation data. Ethically, organoid technology raises important questions regarding long‐term storage, secondary research use, and commercialization. Organoids can be expanded indefinitely, which necessitates transparent consent processes and governance structures [63].

### 7.5 Case Reports and Real‐World Clinical Impact

Individual patient reports and small clinical series provide additional support for the biological relevance of PDO testing. Studies by Vlachogiannis et al. [3], Tiriac et al. [23], de Witte et al. [59], and Kim et al. [60] demonstrated instances in which organoid drug responses paralleled clinical sensitivity or resistance. These findings suggest that PDO testing may help identify ineffective therapies and reveal functional vulnerabilities not captured by genomic profiling alone.

Nevertheless, case reports and observational cohort studies primarily provide hypothesis‐generating evidence. They should not be interpreted as demonstrating clinical benefit, and further prospective interventional studies are necessary to determine whether PDO‐guided treatment decisions improve survival or other patient‐centered outcomes.

### 7.6 Looking Forward

Future progress will depend on continued methodological standardization, multicenter validation, and randomized clinical trials evaluating organoid‐guided treatment strategies. Although accumulating evidence supports the predictive potential of PDOs in selected malignancies, routine clinical implementation will require robust demonstration of analytical validity, clinical validity, and, most importantly, clinical utility. Until such evidence becomes available, PDOs should be regarded as highly promising investigational tools with substantial translational potential rather than established components of standard oncology practice. The sequential evidentiary and logistical gates that determine whether PDO testing can inform a real‐time treatment decision, or remains investigational, are summarized in Figure 2.

**Figure 2:**
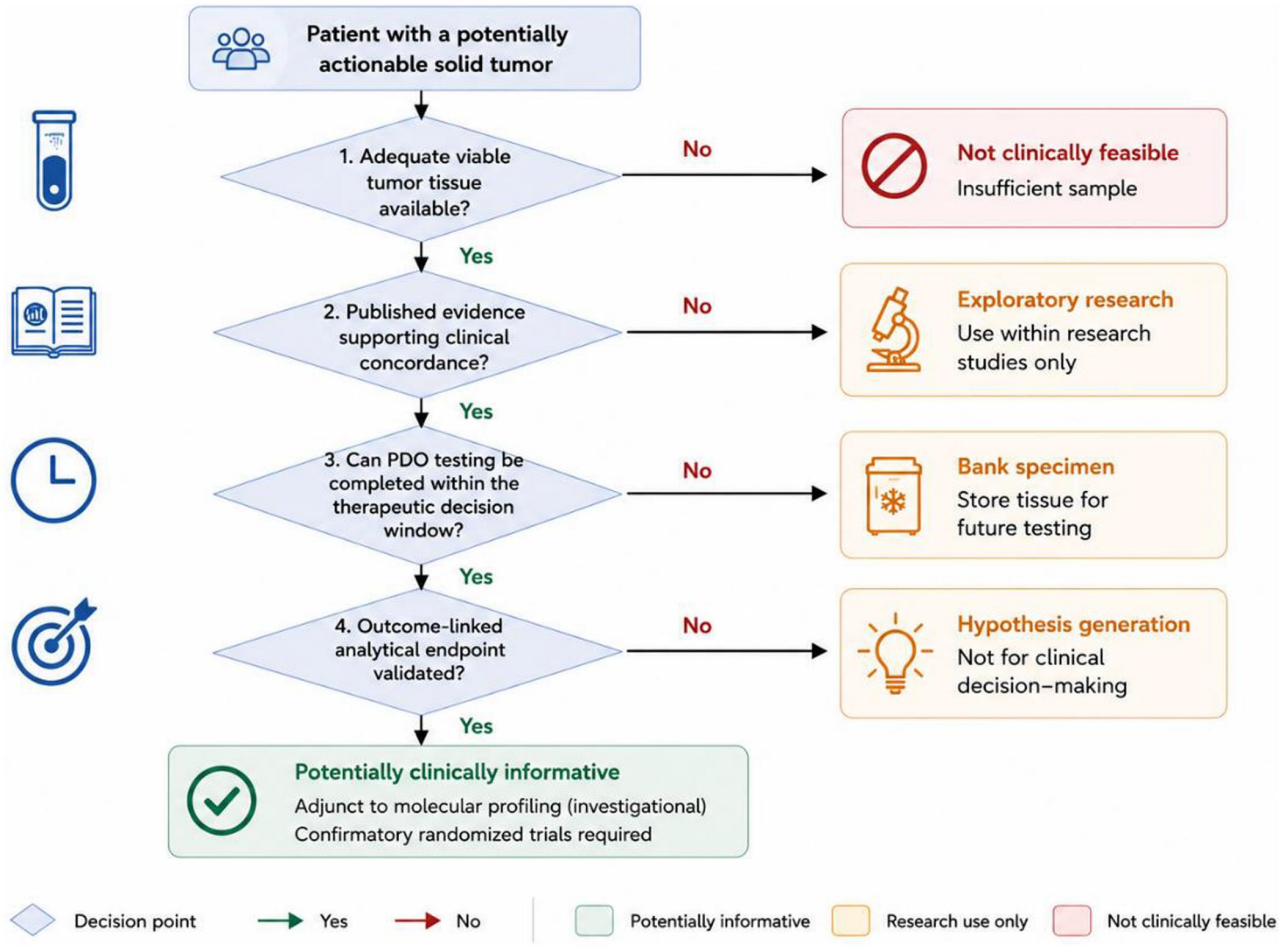
Clinical feasibility decision tree for PDO‐guided therapy selection. Sequential evidentiary and logistical gates determining whether PDO testing can inform a real‐time treatment decision for an individual patient, or remains investigational. Gates reflect constraints discussed in Sections 5.5, 7 and 8: adequacy of viable tumor tissue (surgical resection vs. core‐needle biopsy), availability of indication‐specific prospective concordance data, alignment of organoid turnaround time (typically 4–6 weeks) with the therapeutic decision window, and availability of a validated, outcome‐linked response endpoint rather than an unvalidated in vitro metric. Patients clearing all four gates receive PDO data as an adjunct to genomic profiling, not a standalone treatment determinant, since clinical utility has not been confirmed in a randomized trial for any indication (see Figure 1).

## 8 ⁠Challenges and Limitations

### 8.1 Technical Challenges: Standardization, Reproducibility, and Cost

Despite the numerous experimental advantages offered by organoid systems compared with traditional models, several technical barriers continue to limit their broader application in research and clinical settings (Table 3). One major issue is theabsence of standardized culture protocols. Variability in culture media composition, ECM selection, growth factor supplementation and passaging techniques contributes to interlaboratory differences, thereby reducing reproducibility and limiting comparisons across studies [5, 8, 11]. Furthermore, the widespread use of biologically derived matrices such as Matrigel introduces additional variability because of batch‐to‐batch differences in composition. The financial costs associated with establishing and maintaining PDO cultures also remain substantial owing to the requirement for specialized matrices, recombinant growth factors, dedicated laboratory infrastructure, and technical expertise, making PDO culture considerably more resource‐intensive than conventional two‐dimensional cell culture [64]. Finally, organoid establishment rates vary across tumor types and are influenced by tissue quality, tumor cellularity, and previous treatment exposure, resulting in inconsistent success rates among different clinical specimens.

**Table 3:** Advantages and limitations of patient‐derived organoid (PDO) models in oncology. Comparison of the key strengths and current challenges associated with PDO technology in cancer research and precision medicine, including tumor representation, drug screening capability, clinical translation potential, and technical limitations related to tumor microenvironment modeling and culture complexity.

Aspect	Advantages of PDO models	Limitations/challenges
Tumor representation	Preserve patient‐specific genetic alterations, histological architecture, and tumor heterogeneity, allowing faithful modeling of individual cancers	Some stromal and microenvironmental components are lost during culture, which may limit representation of the full tumor ecosystem
Precision medicine applications	Enable **personalized drug screening** to predict patient response and guide treatment selection	Clinical implementation is still limited by the time required to generate organoids and perform drug testing
Drug discovery and screening	Allow **high‐throughput testing of multiple therapeutic agents**, including targeted therapies and chemotherapies	Drug responses may differ from in vivo conditions due to absence of systemic pharmacokinetics and host factors
Genetic and molecular studies	Facilitate investigation of cancer signaling pathways, gene mutations, and mechanisms of drug resistance	Genetic drift and clonal selection can occur during prolonged culture
Co‐clinical modeling	Can be used alongside patient treatment in **co‐clinical trials** to predict therapeutic outcomes	Not all patient samples successfully generate organoids, resulting in variable establishment success rates
Ethical considerations	Reduce reliance on animal models and enable use of small biopsy samples	Require specialized laboratory infrastructure, expertise, and standardized protocols
Tumor microenvironment research	Can be combined with **immune cells, fibroblasts, or extracellular matrix models** to study tumor–microenvironment interactions	Current co‐culture systems remain technically challenging and may not fully replicate the native tumor microenvironment
CAR‐T and adoptive cell therapy	PDOs retain patient‐specific tumor antigens and can provide a patient‐matched platform for evaluating antigen‐specific cytotoxicity. PDO–immune‐cell co‐cultures can therefore be used to compare CAR‐T or other adoptive cell therapies against individual tumor models before clinical administration	Immune‐cell viability, persistence, infiltration into organoids, effector‐to‐target ratios, and co‐culture conditions remain technically difficult to standardize. These limitations currently constrain rapid clinical deployment despite the potential for personalized cytotoxicity screening
Extracellular matrix and clinical‐grade culture	PDOs can be maintained in extracellular matrices that provide structural and biochemical cues required for three‐dimensional growth. Synthetic or chemically defined hydrogels, including PEG‐ and hyaluronic‐acid‐based systems, offer opportunities for greater compositional control and reproducibility	Matrigel is derived from murine sarcoma and exhibits substantial lot‐to‐lot variability, which can alter organoid growth kinetics and experimental readouts. Its animal origin also presents challenges for GMP‐compliant and xeno‐free clinical translation. Standardized synthetic matrices based on PEG, hyaluronic acid, or other defined biomaterials may provide more reproducible environments for clinical‐grade PDO workflows, although tumor‐type‐specific optimization remains necessary

### 8.2 Biological Limitations: Incomplete Recapitulation of TME

A major biological limitation of conventional PDO cultures is their lack of a fully recapitulated TME. Most organoid cultures primarily consist of epithelial tumor cells and therefore lack essential stromal, vascular, neural, and immune components that contribute to tumor progression and therapeutic response in vivo. Consequently, these models cannot fully reproduce physiological nutrient exchange, angiogenesis, immune surveillance, or stromal‐mediated mechanisms of therapeutic resistance. Diffusion limitations may also result in central necrosis in larger organoids. Consistent with these observations, transcriptomic analyses demonstrate reduced expression of stromal and immune‐related gene signatures in PDOs compared with their corresponding primary tumors [16, 64]. Although advances in co‐culture systems incorporating immune and stromal cells are improving physiological relevance, faithful reconstruction of a functional TME remains a significant challenge.

### 8.3 Clinical Hurdles: Scalability, Turnaround Time, and Patient Accessibility

In order for PDOs to function as real‐time clinical decision‐support tools, the interval between tissue acquisition and generation of actionable results must be compatible with therapeutic decision‐making. Although optimized protocols have reduced turnaround times in selected settings, organoid establishment, expansion, and drug testing generally require several weeks, which may limit clinical applicability for rapidly progressing malignancies. Furthermore, implementation of standardized high‐throughput workflows suitable for routine clinical practice remains challenging. Another important limitation is selection bias. Successful organoid establishment depends on obtaining sufficient viable tumor tissue, which is more readily available from surgical resections than from small biopsy specimens. Consequently, patients with unresectable disease or limited biopsy material may be underrepresented in PDO‐based studies, potentially affecting the generalizability of current evidence.

### 8.4 Regulatory and Validation Challenges: Defining Clinical Endpoints

The path to clinical integration requires overcoming major obstacles in regulation and validation. There is still no consensus on standardized, clinically validated endpoints for PDO‐based assays. Studies employ diverse and non‐uniform metrics—such as IC50, GR50, area under the curve (AUC), or viability at a specific radiation dose—to quantify drug or radiation response, making it difficult to establish robust, universally applicable predictive thresholds. Consequently, despite numerous promising retrospective correlation studies, the definitive predictive value of PDOs must be rigorously established through prospective, multicenter clinical trials where treatment decisions are guided by organoid responses and compared against standard‐of‐care outcomes. Regulatory approval for PDOs as companion diagnostics will also necessitate moving away from animal‐derived components to chemically defined, xeno‐free culture systems that ensure safety, consistency, and compliance with good manufacturing practice standards [65]. Figure 3 consolidates the technical, biological, clinical, and regulatory bottlenecks described above (Sections 8.1, 8.2, 8.3, 8.4) against the pipeline timeline and the therapeutic decision window.

**Figure 3:**
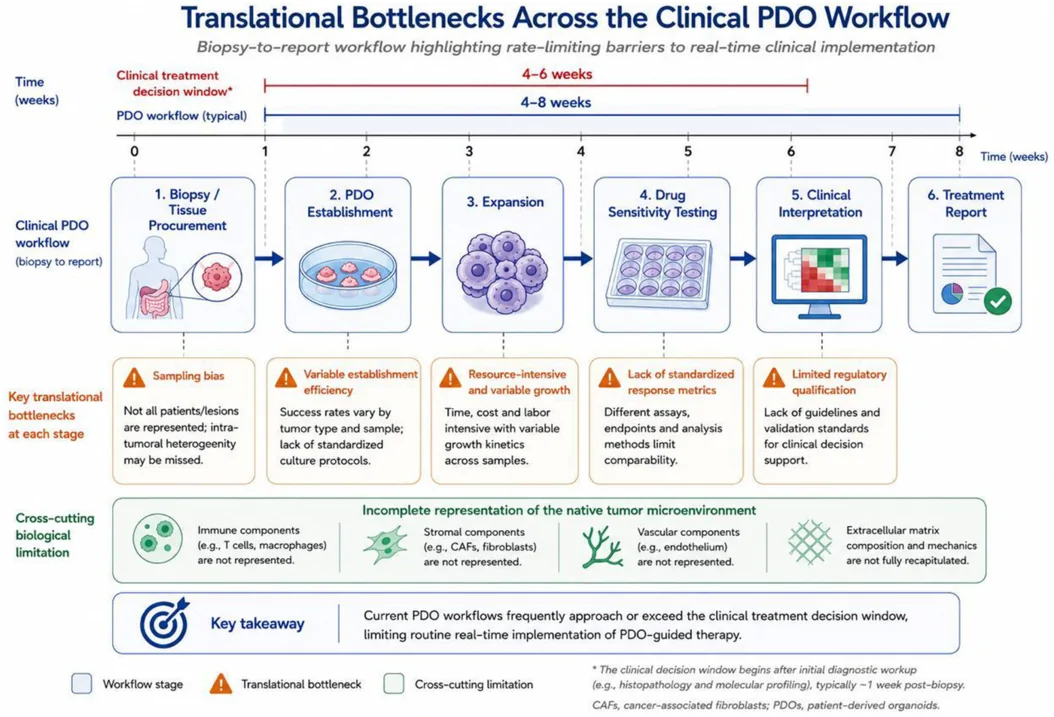
Translational bottlenecks across the PDO testing pipeline. Estimated duration of each stage from tissue procurement to clinician‐facing drug‐sensitivity data, benchmarked against the 4–6 week therapeutic decision window described in Section 8.3, with the principal rate‐limiting constraint annotated at each stage: selection bias toward resection over core‐needle tissue; absence of a standardized culture protocol, including lot‐to‐lot Matrigel variability; cost and cancer‐type–dependent establishment success; lack of a consensus response endpoint (IC50, GR50, AUC, and radiation‐dose viability are used interchangeably); and the regulatory requirement for GMP‐compliant, xeno‐free culture systems before companion‐diagnostic use. A parallel, non‐time‐anchored constraint, loss of immune and stromal gene signatures in epithelial‐only PDOs relative to parent tumor, applies at every stage.

## 9 ⁠Future Directions

Future progress in the PDO field will depend not only on continued technological innovation but also on overcoming several unresolved translational challenges. They provide reliable models for therapy testing tumor heterogeneity capture and treatment response prediction in contrast to traditional systems [66]. According to new research on lung cancer, PDOs can be used to study radiation and drug resistance. These models guide the development of specialized strategies to get past resistance and help pinpoint the molecular reasons why treatments don't work [67]. Furthermore, CRC organoids have been shown to impact immune responses particularly the activation of natural killer (NK) cells. They provide information for optimizing immunotherapy by exposing the ways in which hypoxia and TGF‐β signaling influence patient‐specific immune diversification [68].

Recently, a variety of tumor types such as endometrial, lung and breast cancers have become able to produce PDOs. In clinical practice they are useful tools for oncologists because they more accurately replicate the complexity of patient tumors than 2D cultures [69]. A 2022 study by Yue et al. [70] created a multiple myeloma PDX model that is resistant to bortezomib. It shows how such systems can be modified to investigate drug resistance and aid in the creation of innovative therapeutic approaches.

Precision oncology advancements highlight the use of PDOs in conjunction with biomaterials to produce an environment that more closely mimics the microenvironment of tumors. These integrated systems increase the precision of drug tests and close the gap between experimental treatments and clinical translation [71]. To screen for customized therapy, a high‐throughput 3D glioblastoma model was developed using glycosaminoglycan hydrogels [72]. This platform allows for precise control of ECM cues which enhances the predictive power of organoid‐based drug testing.

Similarly, a functional personalized nano‐therapy for osteosarcoma was developed by researchers through combining different agents that were appropriate for tumor biology of each patient [73]. This approach is consistent with combinatorial therapy screening and PDO‐based personalized treatment strategies.

PDOs also allow the use of liquid biopsy‐derived organoids which enable real‐time evaluation of treatment effectiveness using patient blood samples and non‐invasive tracking of tumor development [74]. Increasingly, the development of patient‐derived models is depending on the explicit fusion of machine learning and artificial intelligence (AI/ML). It is now necessary to use these computational tools in order to process the enormous datasets produced by high‐throughput organoid screening. With the use of AI/ML researchers can more accurately predict drug synergies and spot subtle phenotypic changes than they could with manual analysis. In non‐invasive diagnostics, the trend toward organoids derived from liquid biopsies also represents a paradigm shift. In contrast to traditional tissue biopsies, organoids made from terminal fluids or circulating tumor cells (CTCs) allow for long‐term disease monitoring. With this method physicians can document the dynamic development of a tumor and modify treatments instantly when resistance indicators appear.

Finally, cell and gene therapy is being explicitly and increasingly mentioned as the main use case for these models. Organoids are currently being used as biological testbeds to maximize CAR‐T cell activation and validate CRISPR‐based gene editing [75]. By ensuring that advanced cellular therapies are both safe and effective prior to being reinfused into the patient, the gap between clinical bedside application and genetic engineering is being bridged.

## 10 ⁠Conclusion

Patient‐derived organoids have evolved from innovative experimental models into one of the most promising platforms for investigating human cancer biology. By preserving important architectural, molecular, genetic, and functional characteristics of their tumors of origin, PDOs provide experimental systems that more closely recapitulate patient tumors than conventional two‐dimensional cell culture models. Their ability to retain patient‐specific heterogeneity has made them valuable tools for studying tumor evolution, mechanisms of therapeutic resistance, and functional responses to anticancer agents. The establishment of large organoid biobanks, together with advances in genomic profiling, high‐throughput drug screening, single‐cell sequencing, and CRISPR‐based functional genomics, has further expanded the potential applications of PDOs in translational cancer research and functional precision oncology. Despite these advances, important biological, technical, and clinical challenges remain. Standardization of culture conditions, extracellular matrices, growth factor formulations, and analytical methodologies is required to improve reproducibility across laboratories. Furthermore, most PDO systems lack crucial components of the TME, including immune cells, stromal elements, and vascular networks, all of which influence tumor progression and therapeutic response. Ongoing efforts to integrate organoids with co‐culture systems, microfluidic technologies, and engineered biomaterials are expected to improve the physiological relevance of these models. Current evidence supports the value of PDOs as experimental and translational research platforms, while their role in routine clinical decision‐making continues to be evaluated. Further methodological standardization, multicenter validation, and prospective interventional studies will be required to establish their clinical utility. As these challenges are addressed, PDOs are likely to become increasingly important tools for bridging laboratory research and precision oncology, ultimately contributing to more individualized approaches to cancer treatment.

## Author Contributions

**Abdullah Jabri:** conceptualization, literature review, writing – original draft preparation. **Mohamed Alsharif:** literature review, writing – original draft preparation. **Abdulaziz Mhannayeh:** literature review and manuscript editing, manuscript review and editing. **Bader Taftafa:** literature review and manuscript editing, manuscript review and editing. **Dania Sibai:** literature review and manuscript editing, manuscript review and editing. **Abdulrahman Elsalti:** critical revision of the manuscript and scientific input, writing – original draft preparation. **Raghad Alsharif:** literature review and manuscript editing, writing – original draft preparation. **Tanveer Ahmad Mir:** literature review, writing – original draft preparation. **Mohammed Imran Khan:** literature review, writing – original draft preparation. **Jahan Salma:** literature review, writing – original draft preparation. **Ahmed Yaqinuddin:** conceptualization, supervision, literature review, writing – original draft preparation manuscript review and editing.

## Ethics Statement

This article is a review of previously published literature and did not involve human participants, human tissue, or animal subjects.

## Consent

The authors have nothing to report.

## Conflicts of Interest

The authors declare no conflicts of interest.

## Data Availability

Data sharing is not applicable to this article as no new datasets were generated or analyzed during the preparation of this review.
